# Improved PBFT algorithm for high-frequency trading scenarios of alliance blockchain

**DOI:** 10.1038/s41598-022-08587-1

**Published:** 2022-03-15

**Authors:** Song Tang, Zhiqiang Wang, Jian Jiang, Suli Ge, GaiFang Tan

**Affiliations:** 1 Institute of Applied Mathematics, Hebei Academy of Sciences, No. 46 South Youyi Street, Shijiazhuang, 050081 China; 2 Hebei Authentication Technology Engineering Research Center, No. 46 South Youyi Street, Shijiazhuang, 050081 China; 3 Julu Institute of Applied Technology, Guangming North Street, Xingtai, 055250 China; 4 Talent Exchange Service Center of the Department of Human Resources and Social Security of Hebei Province, No. 9 Yuhua Road, Shijiazhuang, 050001 China

**Keywords:** Computational science, Computer science, Information technology, Software

## Abstract

With the continuous development of blockchain technology, the application scenarios of alliance blockchain are also increasing. The consensus algorithm can achieve distributed consensus among nodes in the network. At present, the practical byzantine fault tolerance algorithm (PBFT) consensus algorithm commonly used in alliance blockchain requires all nodes in the network to participate in the consensus process. Experiments show that when the number of consensus nodes in the system exceeds 100, the bandwidth consumption and consensus delay will greatly increase, resulting in the inability of PBFT to be applied. In scenes with many nodes. How to improve the performance of alliance blockchains safely and efficiently has become an urgent problem to be solved at present. For the PBFT commonly used in alliance blockchains, there are some problems, such as large communication overhead, simple selection of master nodes, and inability to expand and exit nodes dynamically in the network. This paper proposes an improved algorithm tPBFT (trust-based practical Byzantine algorithm), which is suitable for high-frequency trading scenarios of consortium chains. By introducing a trust equity scoring mechanism between nodes in the network, the list of consensus nodes can be dynamically adjusted. tPBFT simplifies the pre-prepare stage of the PBFT consensus process, and realizes the verification of the hash transaction list in the reply stage, thereby reducing the interaction overhead between network nodes. Theoretical analysis and experiments show that when the number of nodes in the network is greater than 30, with the further increase of the number of nodes, the improved tPBFT algorithm has a relatively large performance in terms of node communication overhead, consensus efficiency and scalability outperforms the PBFT algorithm.

## Introduction

Because blockchain technology has the characteristics of decentralization, data security and data tamper-proof^1^, its application value is becoming increasingly prominent. In recent years, the application fields of blockchain not only involve government supervision, finance^2^, copyright protection^3^, supply chains, justice and other fields but also bring new development opportunities for public service fields such as renewable resource recovery and carbon trading. The emergence of Ethereum^4^, superledgers^5,6^ and other platforms has promoted the rapid development of blockchain technology. However, at present, existing blockchain consensus algorithms have the problems of excessive resource consumption and high delay in confirming transactions^7^, which has brought many obstacles to industrial applications. According to the current and future development needs, improving the consensus efficiency in the blockchain is the key step to realize its application and promotion.

Blockchain is a chain structure that organizes and encrypts data according to blocks and then forms according to the generated time sequence. Its tamper-proof and decentralization are maintained by a consensus algorithm^8,9^. In essence, it is a distributed database deployed on many nodes. To ensure the correctness and consistency of data on these nodes, a consensus algorithm plays an important role^10^. At present, the research and development of consensus algorithms is fast, and there are many consensus algorithms. Proof of work (POW)^11^ and proof of stake (POS)^12,13^ are commonly used as consensus algorithms in public chain blockchain, but POW algorithm has the problem of computing power consumption and waste of resources, POS has the tendency of centralization, and the consensus efficiency is still not high enough; in the alliance blockchain, practical Byzantine fault tolerance (PBFT)^14^ and Replicated And Fault Tolerant (raft)^15^ are widely used as consensus algorithms. The traditional PBFT has some disadvantages, such as low efficiency and low practical operability. Although the raft algorithm is efficient, it cannot prevent malicious nodes.

## Related research

In the process of classification and performance improvement evolution of the consensus algorithm, the consensus algorithm mentioned above is representative in the blockchain system. PBFT supporting Byzantine fault tolerance is optimized to continuously improve transaction speed and performance under the condition of ensuring security. It is the first choice of the alliance blockchain. PBFT is an algorithm based on state machine replica replication, which is used to solve the problem of state machine replica consistency in distributed systems^16^ and allows the correct implementation of consensus when the fault node does not exceed the total network node (N-1)/3. The complexity of the traditional Byzantine protocol is reduced from the exponential level to the polynomial level, which makes the application of the Byzantine fault-tolerant algorithm in alliance chains possible. The roles of nodes are defined as master nodes, slave nodes and clients. At the beginning, the master node is randomly selected by the algorithm. Later, after the view switching process, the slave nodes are elected as the master node in turn.

**Figure 1:**
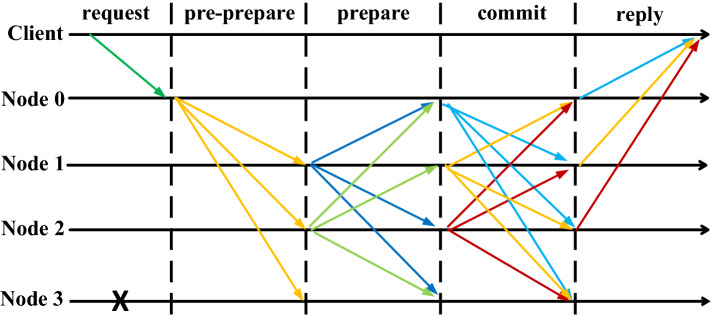
PBFT consensus process.

The consensus protocol (consensus process) of the traditional PBFT algorithm mainly includes the following five communication phases: request, pre-prepare, prepare, commit and reply phases. As shown in Fig. 1, when a transaction needs to be written to the blockchain in the request phase, the client will send a request to master node 0. In the prepare phase, master node 0 forwards the request to slave node 1, slave node 2 and slave node 3. In the preparae phase, each slave node broadcasts its received message to all other nodes. In the commit phase, each node broadcasts a commit message and executes the requests in the transaction list. After verifying the requests in the transaction list and view, in the final reply phase, the node sends the result of responding to the client's request to the client. When the client receives F + 1 identical responses (F is the maximum number of fault tolerant nodes of PBFT), the response is the result of consensus reached by all nodes in the blockchain system.

The PBFT algorithm can solve the Byzantine generals problem, and even if there are a certain number of Byzantine nodes (malicious nodes), a consensus can still be reached among distributed nodes. However, this algorithm requires all nodes in the network to participate in the consensus process. When the number of consensus nodes in the system increases, the bandwidth consumption and consensus delay will greatly increase, so the scale of the consensus cluster becomes the main factor limiting the performance of the PBFT algorithm.

At present, some scholars have studied PBFT algorithm. Lao and l evaluated the performance of the PBFT algorithm implemented by hyperledger fabric^17^, which shows that the consensus efficiency is good when the number of nodes in the network is small, but its performance decreases sharply with the increase in the number of nodes in the network. At present, the optimization of the PBFT algorithm mainly has two aspects: one is to control the number of nodes participating in the consensus, and the other is to optimize the consensus process. Gan Jun^18^ proposed an improved PBFT consensus algorithm ePBFT, which allows nodes to dynamically join and exit by setting the node life cycle and improves the main node selection method of PBFT through the longest chain principle, but it can only be applied to scenes with few nodes. Yanjun Jiang and other authors proposed a high-performance and scalable Byzantine fault tolerance (HSBFT)^19^. First, the algorithm optimizes the communication process of PBFT to reduce its complexity from O(N2) to O(N). At the same time, to make the algorithm dynamically change the number of nodes at runtime, the algorithm introduces a node state table (NST)^19^ but reduces decentralization. Lei, K et al. proposed the right of speech based Byzantine fault tolerance (rbft)^20^ Consensus algorithm: in rbft, more than 2/3 nodes in PBFT need to reach an agreement, which can be changed into nodes that need more than 2/3 voice in the whole system. Although the consensus efficiency is improved, supernodes are allowed to appear for a long time, which will weaken the multicenter characteristics of the alliance blockchain. In addition, Hao, X and others have also optimized the stability of PBFT^21^. This scheme realizes the dynamic joining and exiting of nodes in the cluster but also introduces the centralized management node CA^21^. This blockchain multicenter feature is contrary to and has security risks. Table 1 lists whether the three algorithms of PBFT and the above-mentioned optimized EPBFT, HSBFT, and RBFT support the dynamic increase and decrease of nodes, whether there is centralization, the time complexity of communication interaction, whether they have dynamic evaluation of nodes in the network, etc. Contributions and limitations.

**Table 1:** Comparison of PBFT, EPBFT, HSBFT, RBFT algorithms.

Consensus algorithm	Support node dynamic expansion	Centralized	Time complexity	Dynamic evaluation of nodes
PBFT	No	No	O(n2)	No
EPBFT	Yes	No	O(n2)	No
HSBFT	Yes	Yes	O(n)	No
RBFT	Yes	Yes	O(n2)	Yes

It can be seen from Table 1 that there are few optimizations for the PBFT consensus algorithm, while optimizing the consensus efficiency, the security of the alliance blockchain network, and dynamically considering the properties of the network nodes themselves. Allowing the emergence of super nodes will weaken the multi-center feature of the alliance blockchain. Aiming at the application scenarios of alliance blockchain such as high-frequency trading, this paper designs an efficient consensus mechanism and related algorithms by improving the PBFT consensus algorithm, so as to achieve faster block generation speed and higher transaction throughput than the existing technology, and improve the alliance. Scope of application of blockchain.

## Technical feasibility analysis

### Analysis of existing problems

In the classic PBFT algorithm, nodes need to communicate with each other and needs three-stage broadcasting, so the communication complexity is too high^18^. The random selection of master nodes may select malicious nodes and force re-election, which will affect the execution efficiency of the algorithm. In addition, when the number of nodes is too large, the communication consumption between nodes will greatly increase, exposing problems such as the low communication efficiency of the PBFT algorithm and resulting in low scalability. Generally, the performance of the measured system drops very much when the number of nodes is approximately 100. When PBFT transmits large data packets, the delay is very high when the network is unstable. In the pre-prepare, prepare and commit phases, each node needs to package, verify and broadcast the transaction list to other nodes, which reduces the efficiency and performance of consensus among nodes and puts great pressure on network communication^19,20^. At present, PBFT can only be limited to alliance blockchain or private chain scenarios with few blockchain nodes.

### Propose solutions to existing problems

In view of the above problems in high-frequency trading scenarios, this paper designs a consensus method suitable for the high-frequency transaction scenario of the alliance chain by introducing the trust equity scoring mechanism between the consensus nodes in the alliance chain and improving the Byzantine fault tolerant algorithm^21^. The consensus method is based on the traditional PBFT consensus algorithm and mainly optimizes the existing PBFT algorithm technology, which has a complex message communication mechanism and limited network node data. The consensus nodes cannot exit independently, and the data packet structure is redundant to improve the network communication efficiency and scalability in the consensus process and finally achieve the purpose that the alliance blockchain can be used in high-frequency trading scenarios.

## Design consensus algorithm

### Overview of algorithms

**Figure 2:**
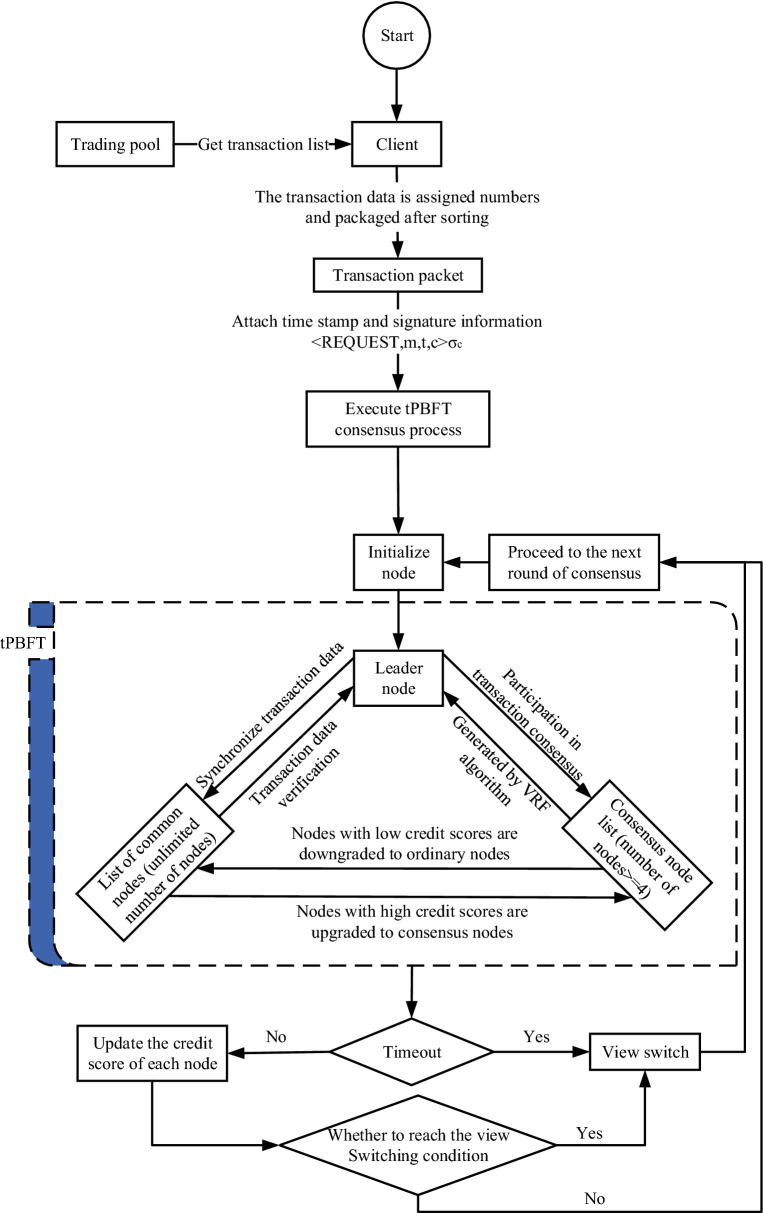
Consensus flow of tPBFT algorithm.

In this paper, a practical Byzantine fault tolerant algorithm based on the Trust Equity Score (tPBFT) is presented for scalable nodes of federated blockchains and high-frequency trading scenarios, as shown in Fig. 2.

Consensus methods suitable for high-frequency trading scenarios in federation chains are mainly implemented by introducing a trust equity scoring mechanism and improving the Byzantine fault tolerance algorithm among consensus nodes in the federation chains. First, the client obtains the transaction list from the transaction pool, sorts the transaction list and packages it as message m, and attaches timestamp T and client signature information σ_c_ to form the REQUEST stage data package <<REQUEST,m,t,c > σ_c_ >. After the leader node receives the message, assign the sequence number n to the request packet, attach the view number V and other information, and package to form a PREPARE phase message package <<PREPARE, v, n, d(m) > σ_P_, m >, where d(m) is the summary of message m and σ_c_ is the primary node signature.

The nodes in the system are divided into two roles by the trust equity scoring mechanism: consensus nodes participating in consensus and common nodes participating in storage validation. During the confirmation phase, the leader node will <<PREPARE, v, n, d(m) > σ_P_, m > be distributed to consensus nodes, which agree on the transaction list and form COMMIT phase packets <<COMMIT, v, n, D(m), i > σ_i_ >, where I represents the i-th block chain node in the network, σ_i_ is the signature information for the i-th node. In the submission phase, the leader node collects up to 2f + 1 nodes (f is the maximum number of fault tolerant nodes in the network) and sends a confirmation packet, which proves that the transaction is valid and packages the REPLY phase packets <<REPLY, v, t, p > σ_P_, m >, where p is the primary node, data packages are distributed to normal nodes, and valid confirmation replies are made to clients for transactions.

The traditional PBFT algorithm is simplified into three phases: PREPARE, COMMIT and REPLY. In the COMMIT and REPLY phases, after the improvement of PBFT by the tPBFT algorithm we designed, the transaction list in the packet only contains the Hash list of transaction hashes.

### Establishing a trust equity scoring mechanism

The PBFT algorithm can solve the general Byzantine problem; that is, the distributed nodes can still reach a consensus when malicious nodes are allowed. However, the algorithm requires all nodes to participate in the consensus. As the number of nodes in the network increases, the consensus delay and network bandwidth consumption will increase dramatically, so the number of nodes in the network is a key factor that restricts the performance of the PBFT algorithm.

**Figure 3:**
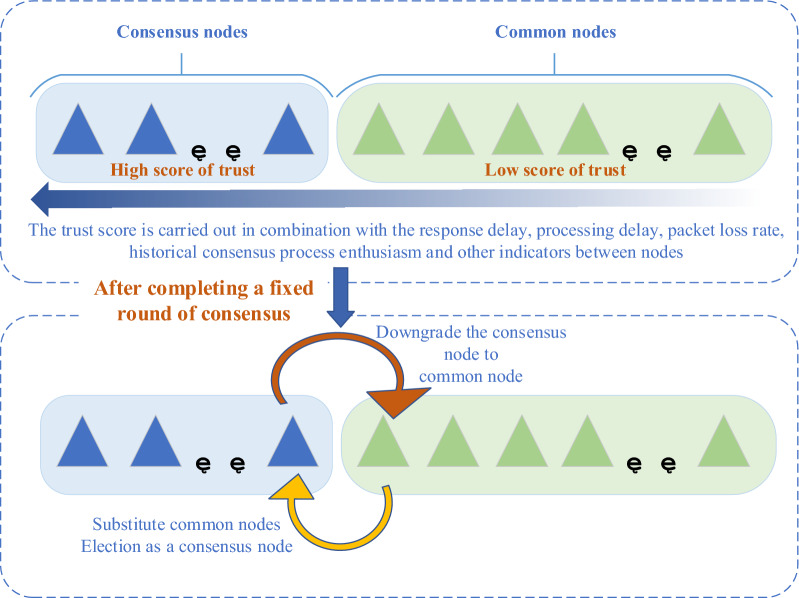
Node trust equity scoring model.

This paper establishes a trust model to score nodes in the network, periodically demotes the consensus nodes with lower scores, and the common nodes with higher scores can be elected as consensus nodes to participate in consensus accounting by substitution. The improvement of the Byzantine fault tolerance algorithm applied to extensible network nodes mainly reflects the increased dynamics and reliability of the network. The process is shown in Fig. 3 below:

The blockchain is essentially a distributed database deployed on many nodes. In order to ensure the correctness and consistency of the data on these nodes, the consensus algorithm plays an important role. Existing consensus algorithms usually do not take into account the different bandwidth overhead and network delay of each node in the network, resulting in nodes participating in the consensus process in the network, and when data synchronization between nodes is performed, most nodes wait for a long time for a few nodes with high network delay. The current situation makes it difficult to meet the performance requirements of blockchain technology in high-frequency trading scenarios. Therefore, this paper combines the response delay, processing delay, packet loss rate, historical node availability and reliability among nodes in the network, all nodes will score their trust every time they complete a transaction. After completing a fixed round of transactions, the nodes with low trust scores will be degraded to ordinary nodes, and the ordinary nodes with high scores can be elected as consensus nodes. At the same time, the historical trust scores of all consensus nodes are cleared and restarted. Based on the trust equity scoring mechanism, it ensures that the consensus node has the highest trust score. When a blockchain node has low response delay and low processing delay, it shows that the node has strong processing capacity. At the same time, the historical availability and reliability indicators of the node are introduced to reduce the risk of random selection of the master node and consensus node of the original PBFT algorithm, improving network security.

Because the trust equity score includes multiple dimension indicators, some indicators are high-quality (the higher the indicator, the better, such as historical node availability, reliability, throughput, etc.), and some indicators are low-quality (the lower the indicator value, the better, such as response delay, processing delay, packet loss rate, etc.). Therefore, a unified normalization method is established for high and excellent indexes, as shown in the formula (1):

1 $${\mathrm{Y}}_{\mathrm{ij}}=\frac{{ -\mathrm{ y}}_{\mathrm{j min}}}{{\mathrm{y}}_{\mathrm{j max}} - {\mathrm{ y}}_{\mathrm{j min}}}$$

In the formula (1), Y_ij_ is the actual measured value of the i-th node in the j-th high optimization index; ${\mathrm{y}}_{\mathrm{j max}}$ is the actual measured maximum value of all nodes of the j-th high optimization index;${\mathrm{y}}_{\mathrm{j min}}$ is the actual measured minimum value of all nodes of the j-th high optimization index.

Establish a unified normalization method for low and excellent indicators, as shown in the formula (2):

2 $${\mathrm{Y}}_{\mathrm{ik}}=1- \frac{{ {\mathrm{y}}_{\mathrm{ik}} -\mathrm{ y}}_{\mathrm{k min}}}{{\mathrm{y}}_{\mathrm{k max}} - {\mathrm{ y}}_{\mathrm{k min}}}=\frac{{{\mathrm{y}}_{\mathrm{k max}} -\mathrm{ y}}_{\mathrm{ik}} }{{\mathrm{y}}_{\mathrm{k max}} - {\mathrm{ y}}_{\mathrm{k min}}}$$

In the formula (2), Y_ik_ is the actual measured value of the i-th node in the k-th low optimal index; ${\mathrm{y}}_{\mathrm{k max}}$ is the actual measured maximum value of all nodes of the k-th low optimal index;${\mathrm{y}}_{\mathrm{k min}}$ is the actual measured minimum value of all nodes of the k-th low optimal index.

Assuming that the number of high-quality indicators and low-quality indicators in multiple dimensions of the trust equity score is M1 and M2, the index information of high-quality and low-quality dimensions is fused and weighted to obtain the trust equity score $\mathrm{Q}({\mathrm{n}}_{\mathrm{i}})$ of the ith node in the network, as shown in the formula (3):

3 $$\mathrm{Q}({\mathrm{n}}_{\mathrm{i}})=\sum_{\mathrm{j}=1}^{\mathrm{m}1}\left(\frac{{ {\mathrm{y}}_{\mathrm{ij}} -\mathrm{ y}}_{\mathrm{j min}}}{{\mathrm{y}}_{\mathrm{j max}} - {\mathrm{ y}}_{\mathrm{j min}}}\right)\times {\mathrm{w}}_{\mathrm{j}} +\sum_{\mathrm{k}=1}^{\mathrm{m}2}\left(\frac{{{\mathrm{y}}_{\mathrm{k max}} -\mathrm{ y}}_{\mathrm{ik}} }{{\mathrm{y}}_{\mathrm{k max}} - {\mathrm{ y}}_{\mathrm{k min}}}\right)\times {\mathrm{w}}_{\mathrm{k}}$$

In the formula (3): i $\in [\mathrm{i},\mathrm{n}]$.

### Determine the classification and responsibilities of nodes in the network

In our design of the consensus algorithm tPBFT, network nodes are divided into consensus nodes and ordinary nodes. The consensus node participates in the block consensus of each node in the network and is responsible for receiving and signing the transactions sent by the client, analyzing the transaction content in combination with the status data of its own node, supervising and managing the transactions submitted by the client to prevent evil. The common node receives the data synchronization of the consensus node for signature verification and storage. After the consensus node is downgraded to the common node, the common node with a higher score can be elected as the consensus node.

### Election of consensus nodes in networks

The number of consensus nodes is a configurable parameter. The tPBFT algorithm requires the number of participating consensus nodes C in the network to be expressed as: C ≥ 3F + 1, where C represents the number of consensus nodes, and F represents the number of malicious nodes in the consensus node list. At the same time, honest nodes have the initiative in the consensus of network nodes. Therefore, in order to ensure security and fault tolerance, the number of consensus nodes in the actual production environment should be configured as an integer greater than or equal to 4. If the blockchain network is an alliance chain composed of trusted nodes or requires a high data link rate, the number of consensus nodes can be configured to be smaller to improve the consensus efficiency among nodes. If it is applied in the scenario of a public chain or low reliability between nodes, the number of consensus nodes can be configured to be larger to prevent evil nodes and fault nodes and improve the fault tolerance of the system. Consensus nodes and ordinary nodes can change each other under certain conditions, which solves the problem that the existing PBFT algorithm does not support the addition and exit of new nodes and improves the scalability of network nodes.

### Elect leader node

The consensus nodes elected by the trust equity model in the network have an equal opportunity to compete for the leader node. Each consensus node uses the verifiable random function VRF algorithm to generate random numbers, which are verified with the random numbers generated in the system. The nodes with the same random number are as the leader node of this round of block generation, the leader node is responsible for receiving the transaction packaged by the client, and after signing it, it is distributed to the rest of the nodes in the network.

### Consensus execution process

**Figure 4:**
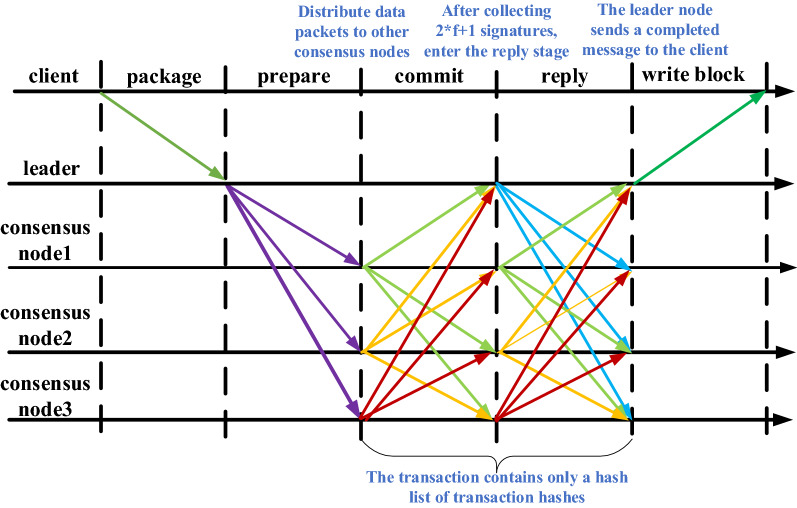
tPBFT consensus execution flow chart.

After the leader node receives the packaged transaction from the client, the tPBFT consensus execution process is divided into three steps: prepare, commit, and reply, as shown in Fig. 4.

1. First, the client obtains the transaction list from the transaction pool, sorts the transaction data and packs it into message m, and attaches timestamp t and client signature information σ_c_ to form the REQUEST phase data package <<REQUEST,m,t,c > σ_c_ >;
2. After receiving the message, the leader node assigns the serial number n to the request data packet and attaches the view number v and other information to form the prepare stage message packet <<prepare, v, n, d (m) > σ_p_,m >, broadcast to other consensus nodes in other networks, where d (m) is the summary of message m, σ_P_ is the signature information of the primary node.
3. After receiving the data packet <<prepare, v, n, d (m) > σ_p_,m> sent by the leader node, other consensus nodes in the network verify the request content and transaction list order in the view. After passing the verification, they enter the commit phase and broadcast the commit phase data packet <<COMMIT,v,n,d(m),i > σ_i_> to other consensus nodes, where I represents the i-th blockchain node in the network, σ_i_ is the signature information of the i-th node;
4. Each node in the network collects the commit broadcast data packets sent by 2F + 1 (F is the maximum number of fault tolerant nodes in the network) and enters the reply stage after legal verification, indicating that it has agreed to all transactions in the transaction list. At the same time, package the reply stage data packet <<reply, v,i, d (m) > σ_i_> and broadcast it to other consensus nodes in the network.
5. In the REPLY stage, the full verification of the transaction list in the traditional PBFT is converted into the verification of the Hash value, thereby reducing the amount of data transmission. After receiving more than 2F + 1 replies, the leader node will prove that the transaction is valid and write the transaction into the local ledger. If the intermediate process fails to pass the verification, the consensus node will broadcast the view change message, switch the view, and re elect the leader node.
6. Finally, the leader node distributes the agreed packets to ordinary nodes and clients.

### Commit and reply phases based on transaction hash

In order to facilitate the verification of the transaction list, the traditional PBFT algorithm contains the same data packets in the commit and reply phases, resulting in repeated transmission of the transaction list. After the tPBFT algorithm we designed improves the PBFT, the transaction list in the data packet only contains the hash list of the transaction hash, and the authenticity of the transaction can be confirmed only by the Hash check. A packet of several MB, after optimization, a transaction state becomes a few hundred bytes. In the reply phase, the nodes participating in the consensus of the network already have all the data of the transaction list. Therefore, the full verification of the transaction list in the traditional PBFT in the reply phase can be converted into the verification of the hash value, thereby reducing the amount of data transmission. And the Hash value is signed with the sender's digital certificate to ensure that only the hash list is verified in the reply phase is safe for the consensus algorithm, and the third party cannot obtain the sender's private key, so data forgery cannot be performed. Ultimately, it is guaranteed that each node of the alliance blockchain will store the requests from the client into the blockchain in the same order of transaction list.

## Analysis and experiment

Assuming that the total number of nodes in the network is N (N ≥ 4), and the number of consensus nodes is C (N ≥ C, C ≥ 4). Since the tPBFT algorithm proposed in this paper selects some nodes to participate in the consensus process through election, it is not suitable for public blockchain networks, because there may be many malicious nodes in such blockchain networks, and with as the proportion of malicious nodes increases, malicious transactions may occur. Therefore, the actual application scenario of this paper is the alliance blockchain for high-frequency trading. This kind of blockchain network with permission has a high degree of trust between nodes. Based on the Go language, this paper realizes the simulation of the algorithm flow of tPBFT and PBFT and compares it from the two aspects of network communication overhead and consensus efficiency.

The experimental environment is an Intel(R) Core(TM) i7-10510U CPU 1.80 GHz, memory 16.0 GB, a win10 64-bit operating system, and an Apache-JMeter testing tool.

### Determine the proportion of consensus nodes and ordinary nodes in the network

**Figure 5:**
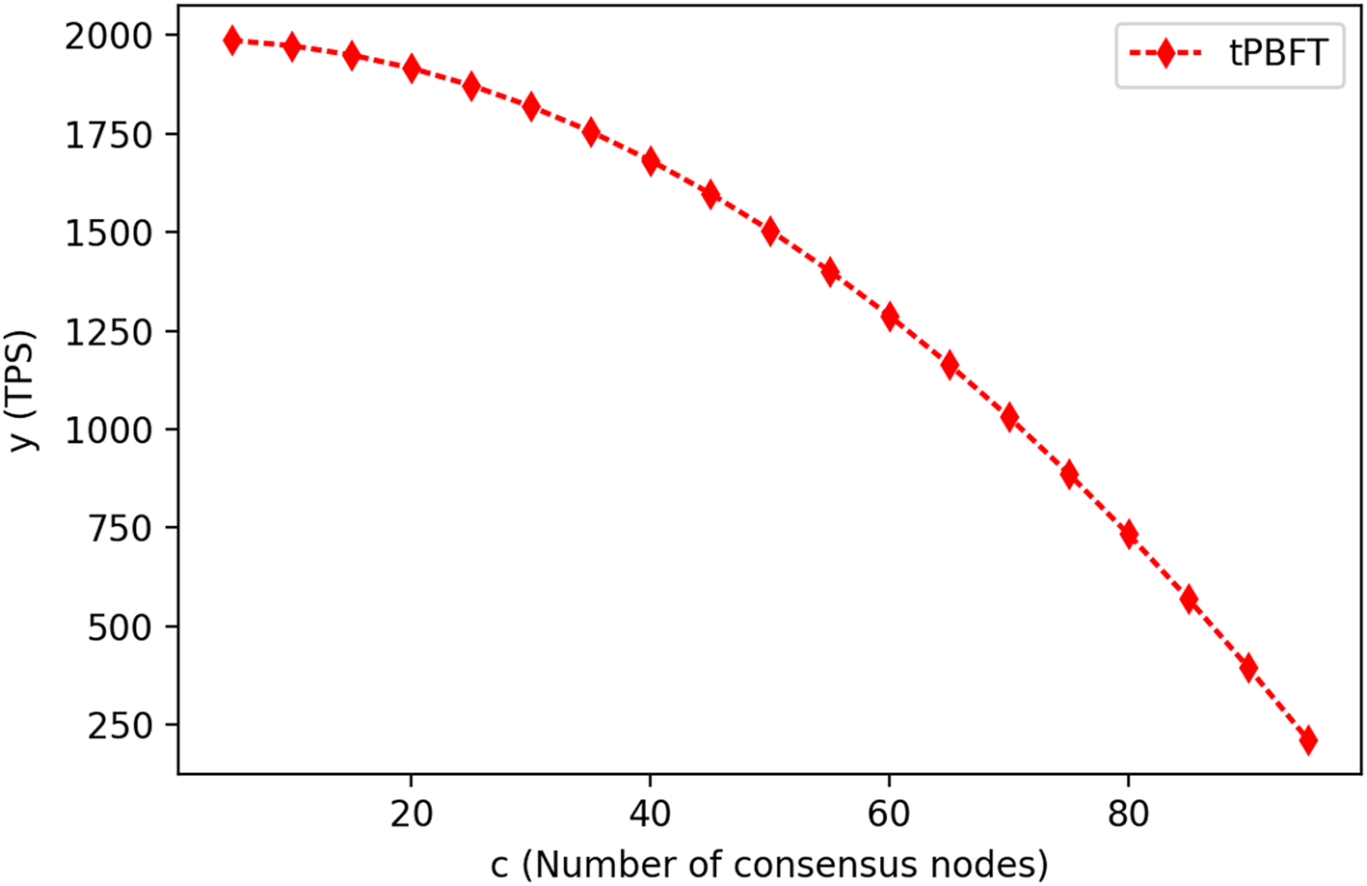
Graph of changes in consensus transaction volume per second when the number of consensus nodes increases.

As the proportion of consensus nodes in the network increases, it takes more time for nodes to reach a consensus on transactions, resulting in a decrease in the number of transactions completed per second. According to the actual measurement, without considering the existence of malicious nodes in the network, the number of nodes in the network is 100, of which the number of consensus nodes is C, and C is 5, 10, 15, 20, 25, 30, 35, 40, 45, 50, 55, 60, 65, 70, 75, 80, 85, 90 and 95. The number of ordinary nodes is 100-c. For each combination of consensus nodes and common nodes, each group conducts 1 million transaction tests and then calculates the number of transactions completed per second (TPS). The results are shown in Fig. 5 below.

When the number of consensus nodes in the network is 5 and the number of ordinary nodes is 95, the TPS is 1960. Then, with the increase in the number of consensus nodes in the network, the TPS gradually decreases. When the number of consensus nodes in the network is 90 and the number of ordinary nodes is 10, the TPS is reduced to 409. It can be seen that without considering the existence of malicious nodes in the network, the smaller the proportion of consensus nodes, the greater the TPS, and the greater the volume of consensus transactions completed per second. Then, in the actual production environment, to select the proportion of consensus nodes, we need to consider the existence of malicious nodes in network nodes.

The tPBFT algorithm requires that the number of nodes participating in consensus in network C be expressed as C ≥ 3FC + 1, where C is the number of consensus nodes and FC is the number of evil nodes in the consensus node list. At this time, honest nodes take the initiative in the consensus of network nodes.

**Figure 6:**
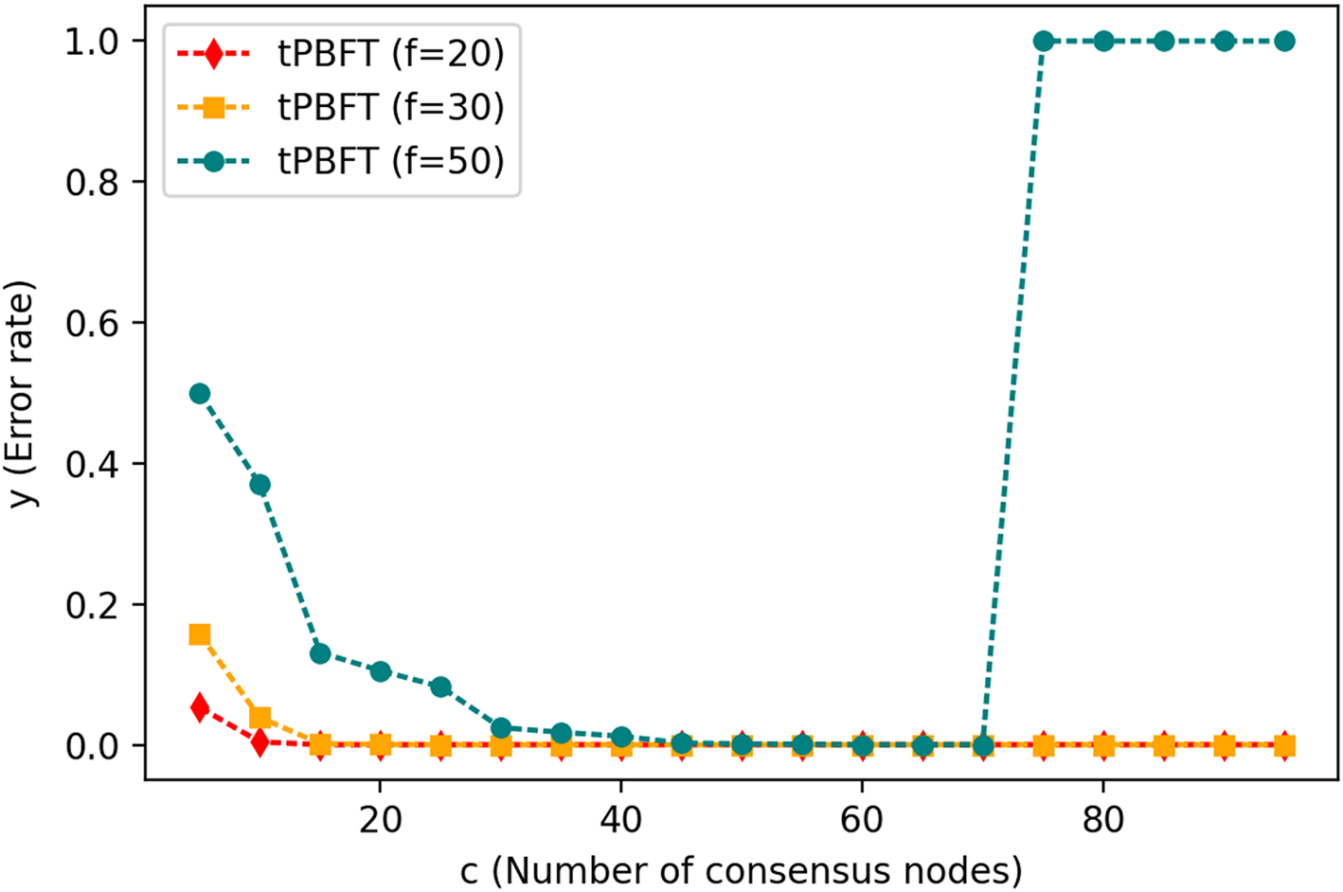
Change chart of consensus transaction volume per second when the number of consensus nodes increases.

When C < 2FC + 1, the evil node takes the initiative in the network node consensus, which endangers the security of the whole network system. According to the actual measurement, the total number of nodes in the network is 100. Considering that there are 20, 30 and 50 malicious nodes and 80, 70 and 50 honest nodes in the network, the consensus node list selects C from malicious nodes and honest nodes according to the trust interest scoring rules, the number of consensus nodes is from 5 to 95, and the number of ordinary nodes is 100 − C. For each combination of consensus nodes and ordinary nodes, the number of honest nodes sends correct transactions, and the number of malicious nodes sends wrong transactions. Each group carries out 100,000 transaction tests and then obtains the number of incorrect transactions. The results are shown in Fig. 6 below.

As seen in Fig. 6, when there are malicious nodes in the network, when the number of consensus nodes C is 30–70, the error transaction rate is the lowest and the fault tolerance rate is the highest. However, an increase in the number of consensus nodes will reduce the TPS. Therefore, when the number of nodes in the network is 100, the value of C is 30–50, which can ensure that the network system has sufficiently high security and a high TPS.

### Analysis of the amount of network communication

1. Analysis of PBFT algorithm network communication interaction
  The implementation principle of the PBFT algorithm is based on the information exchange between all nodes in the network. It is divided into five stages: request, prepare, prepare, commit and reply. The core stage is the last four stages. Assuming that the number of nodes in the network is N (N ≥ 4), in the prepare stage, the master node sends broadcast messages to other nodes, so the communication times are N − 1. In the prepare stage, each node needs to interact with each other to broadcast messages, and the communication times are N(N − 1). In the commit phase, each node also needs to communicate and interact with the commit message in pairs, and the communication times are N(N − 1). In the reply phase, each node needs to send a confirmation message to the client, and the communication times are N. In conclusion, the PBFT algorithm is implemented once, and the communication times are 2N^2^ − 1.
2. Analysis of tPBFT algorithm network communication interaction

The tPBFT algorithm divides the nodes in the network into consensus nodes and ordinary nodes. Only consensus nodes participate in the consensus process. Ordinary nodes synchronize and verify messages after consensus. Assuming that the total number of nodes in the network is N (N ≥ 4) and the number of consensus nodes is C (N ≥ C, C ≥ 4), the number of ordinary nodes is N–C. At the same time, the algorithm simplifies the core stage of the consensus process to prepare Commits and replies. In the preparation stage, the main node broadcasts the packaged message to other consensus nodes, and the communication times are C − 1. In the commit phase, except for the leader node, the remaining C − 1 consensus nodes need to conduct message interaction confirmation, and the communication times are (C − 1)*(C − 1). In the reply phase, each node needs to broadcast the reply message interactively in pairs, and the communication times are C*(C − 1). Finally, the consensus process also needs to send the confirmed transaction message to the client and synchronize it to N − C ordinary nodes for message verification. The communication times are N − C + 1. In summary, the tPBFT algorithm is implemented once, and the communication times are N + 2C^2^ − 3C + 1.

**Figure 7:**
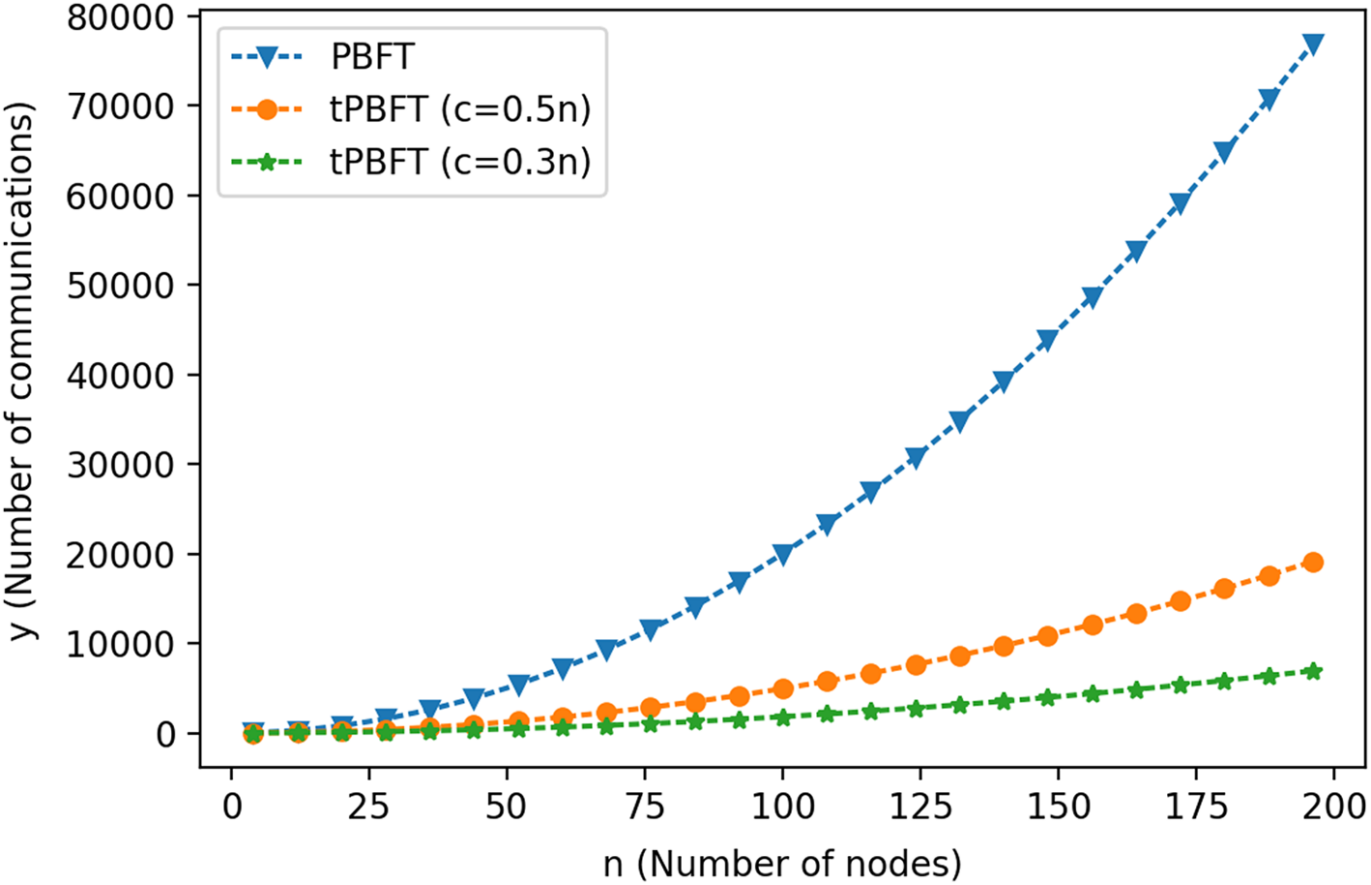
Comparison of communication times between tPBFT and PBFT algorithms.

According to the experimental analysis, the number of network nodes n increases from 4 to 200. For the number of consensus nodes of the tPBFT algorithm, it can be seen from 5.1 that C takes 0.3n and 0.5 N, respectively. The comparison of communication times between the tPBFT and PBFT consensus algorithms is shown in Fig. 7.

In summary, the PBFT algorithm completes a round of consensus processes with communication times of 2N^2^ − 1 and time complexity of O(N^2^). The tPBFT algorithm completes a round of consensus processes with total communication times of N + 2C^2^ − 3C + 1 and time complexity of O(C^2^). With the increase in the number of nodes added to the network, the communication times required by PBFT are significantly higher than those required by tPBFT. In the actual production environment, the consensus efficiency of the alliance blockchain network can be dynamically adjusted by adjusting the number of consensus nodes C in the network.

The comparison between PBFT and tPBFT algorithms is shown in Table 2:

**Table 2:** Comparison of PBFT and tPBFT algorithms.

Algorithm name	Total communication times	Communication time complexity	Support node dynamic exit
PBFT	2N2-1	O(N2)	No
tPBFT	N + 2C2 − 3C + 1	O(C2)	Yes

Assuming that the total number of nodes in the network is N (N ≥ 4), and the number of consensus nodes is C (N ≥ C, C ≥ 4), combined with the above calculation process and Table 2, for the traditional PBFT algorithm and the tPBFT proposed in this paper, respectively The number of network communication and the time complexity of communication are demonstrated on both sides.

First, the number of network communications is demonstrated. The number of network communications for PBFT is 2N^2^ − 1, and the number of network communications for tPBFT proposed in this paper is N + 2C^2^ − 3C + 1.

When N ≥ C, C ≥ 4,

4 $$ {\text{2N}}^{{2}} - {1} > {\text{N}} + {\text{2N}}^{{2}} - {\text{3N}} + {1} \ge {\text{ N}} + {\text{2C}}^{{2}} - {\text{3C}} + {1} $$

It can be seen from formula (4) that the tPBFT algorithm proposed in this paper requires less network communication times than the traditional PBFT algorithm.

Then demonstrate the communication time complexity, the communication time complexity of PBFT is O(N^2^), and the communication time complexity of tPBFT proposed in this paper is O(C^2^).

When N ≥ C, C ≥ 4,

5 $$ {\text{O}}\left( {{\text{N}}^{{2}} } \right) > {\text{O}}\left( {{\text{C}}^{{2}} } \right) $$

It can be seen from formula (5) that the tPBFT algorithm proposed in this paper is better than the traditional PBFT algorithm in the communication time complexity.

### Analyze the efficiency of consensus algorithm

**Figure 8:**
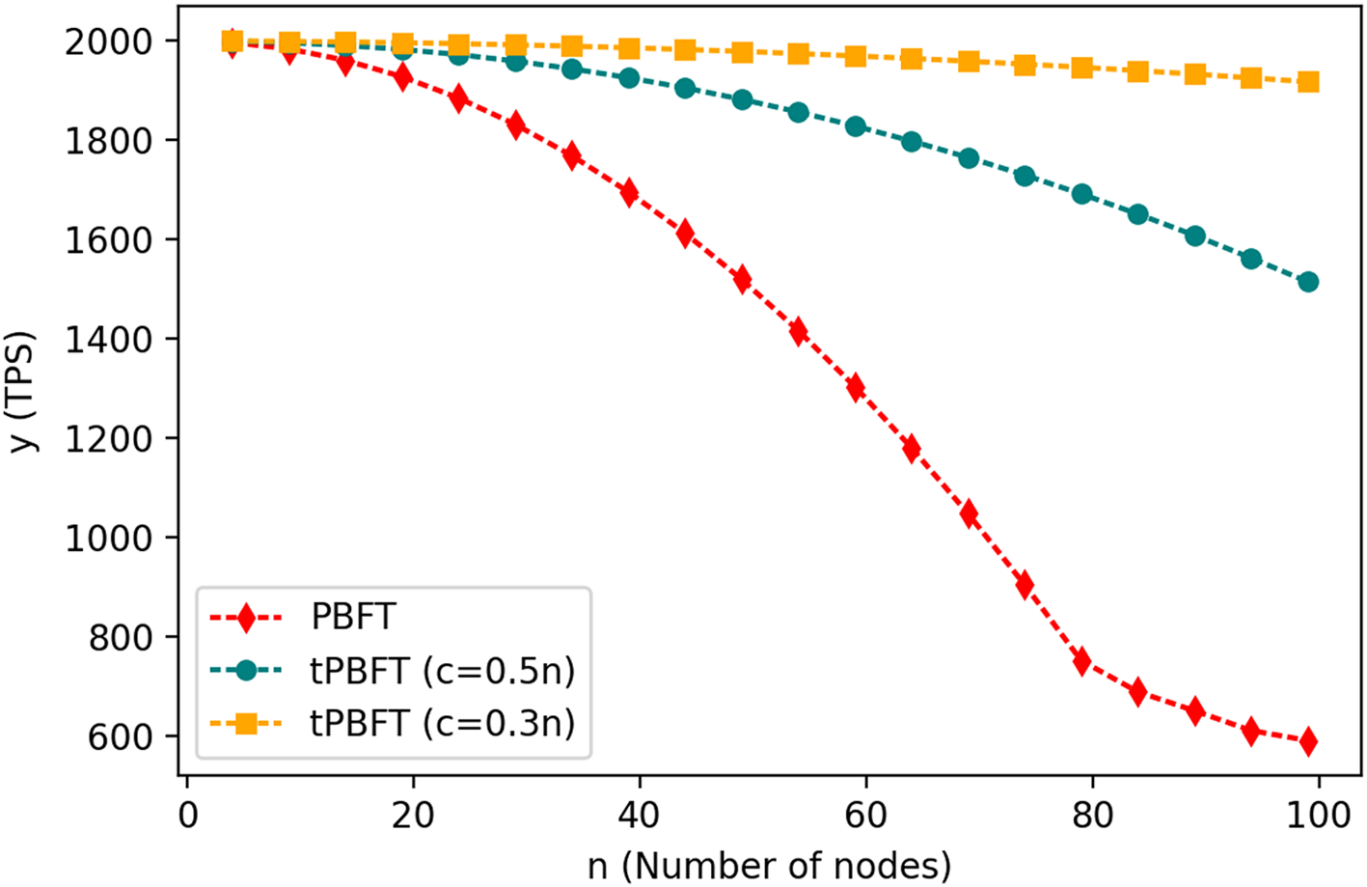
Comparison of TPS between tPBFT and PBFT algorithm.

The consensus efficiency of the alliance blockchain can be measured by the number of transactions completed per second (TPS). The size of the TPS reflects the system's ability to process transactions. The experiments tested the changes of PBFT and tPBFT algorithms with the increase of the number of nodes when the number of nodes N is 4, 9, 14, 19, 24, 29, 34, 39, 44, 49, 54, 59, 64, 69, 74, 79, 84, 89, 94, 99 and C is 0.3 N and 0.5 N:

As the PBFT consensus algorithm involves all nodes in the consensus process, when the number of nodes in the network increases considerably, the communication pressure will be very large, and the consensus delay will increase greatly. As shown in Fig. 8 above, when the number of nodes in the network is greater than 60, the TPS is reduced by almost 50%, and when the network exceeds 100 nodes, the TPS is reduced by 90% compared with 4 nodes. On a year-on-year basis, when the number of nodes of the tPBFT algorithm is 60, the TPS of the system with C values of 0.5 N and 0.2 N decreases only slightly. When the number of nodes exceeds 100, the TPS of the system with a C value of 0.2 N still changes little, and the TPS of the system with a C value of 0.5 N decreases by only 20% compared with the four nodes.

The tPBFT optimizes PBFT algorithm in three aspects: leader node election, transaction list hash and consensus node selection, so that tPBFT also maintains high TPS when there are a large number of nodes, which is more suitable for high-frequency transactions and scalable network node scenarios, so that the alliance blockchain can be popularized and applied in more fields.

## Summary

Based on the PBFT algorithm, this paper proposes an optimized consensus algorithm, tPBFT, suitable for scalable nodes of alliance blockchain and high-frequency transaction scenarios, which is widely used in public service fields such as carbon trading, government supervision, supply chain services, and intelligent inspection. By introducing the trust equity scoring mechanism and improving the Byzantine fault tolerance algorithm among the consensus nodes in the alliance blockchain, the message communication mechanism of the PBFT algorithm is simplified, which can support more nodes and solve the problems of consensus nodes unable to exit independently and redundant packet structures.

However, since the tPBFT algorithm model proposed in this paper does not involve all nodes in the blockchain network participating in the consensus, it is suitable for application in the alliance blockchain, and this kind of network with a high degree of trust between nodes will have better results. The experimental results show that when the number of nodes in the alliance blockchain network is less than 30, the tPBFT and PBFT algorithms do not significantly improve the consensus efficiency. When the number of nodes in the network is greater than 30, with the further increase of the number of nodes, tPBFT is significantly better than the PBFT algorithm in terms of node communication overhead, consensus efficiency and scalability. Next, combined with the technical characteristics of alliance blockchain, we will study the distributed parallel consensus and further improve the model to achieve higher consensus efficiency.
